# CD73-Positive Small Extracellular Vesicles Derived From Umbilical Cord Mesenchymal Stem Cells Promote the Proliferation and Migration of Pediatric Urethral Smooth Muscle Cells Through Adenosine Pathway

**DOI:** 10.3389/fbioe.2022.895998

**Published:** 2022-04-27

**Authors:** Shilin Zhang, Jierong Li, Chunjing Li, Xumin Xie, Jun He, Fengsheng Ling, Bowei Li, Huayan Wu, Zhilin Li, Jianwei Zheng

**Affiliations:** Department of Urology, Affiliated Foshan Maternity and Child Healthcare Hospital, Southern Medical University, Foshan, China

**Keywords:** mesenchymal stem cells, small extracellular vesicles, smooth muscle cells, hypospadias, CD73, proliferation, migration

## Abstract

Smooth muscle cells (SMCs) are the main functional component of urethral tissue, but are difficult to proliferate *in vitro*. Mesenchymal stem cells (MSCs) and mesenchymal stem cell-derived small extracellular vesicles (MSC-sEV) have been shown to promote tissue repair by regulating the proliferation and migration of different types of cells. In this study, we investigated the effect of umbilical cord mesenchymal stem cell-derived sEV (UCMSC-sEV) on the proliferation and migration of pediatric urethral smooth muscle cells (PUSMCs) and the mechanism by which sEV regulates the function of PUSMCs. We observed that UCMSC-sEV can significantly promote the proliferation and migration of PUSMCs *in vitro*. UCMSC-sEV exerted proliferation and migration promotion effects by carrying the CD73 to PUSMCs and catalyzing the production of adenosine. Conversely, the effect of UCMSC-sEV on the proliferation and migration of PUSMCs were no longer observed with addition of the PSB12379 as a CD73 inhibitor. It was found that the phosphatidylinositol 3-kinase (PI3K)/protein kinase B (AKT) signaling pathway in PUSMCs was activated by adenosine or UCMSC-sEV intervention. In summary, UCMSC-sEV promoted proliferation and migration of PUSMCs *in vitro* by activating CD73/adenosine signaling axis and downstream PI3K/AKT pathway. Thus, we concluded that UCMSC-sEV may be suggested as a new solution strategy for the urethral tissue repair.

## Graphical Abstract

Graphical diagram of CD73/Adenosine/PI3K/AKT axis in facilitating cell proliferation and migration of PUSMCs *in vitro*.
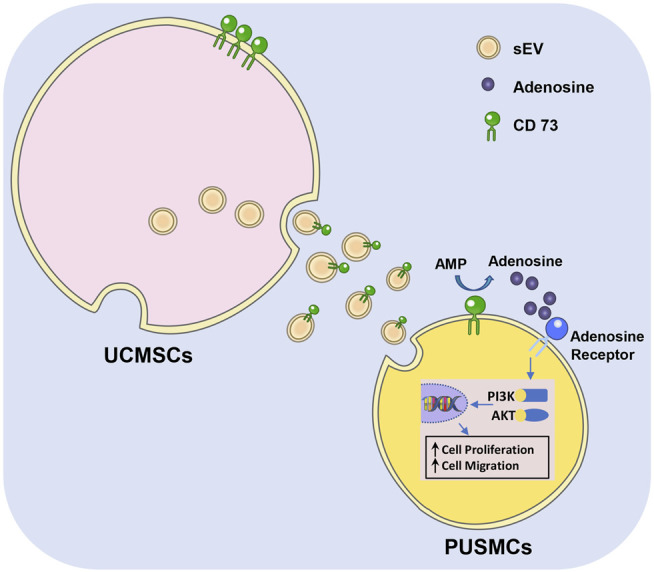

## Introduction

Hypospadias is one of the most common developmental malformation of reproductive organs in male children (Chan et al., 2020) and surgery remains the only treatment in clinical. However, the surgery is associated with postoperative complications such as urinary fistula and penile curvature, mainly due to the lack of protection by smooth muscle cells (SMCs) in the formed urethra (Arenas et al., 2014; Abbas et al., 2017). Smooth muscle is the main functional component of the urethra, and SMCs are terminally differentiated cells. Once damaged, smooth muscle is difficult to regenerate (Sergeant et al., 2019). Some researchers have attempted to obtain primary SMCs and then proliferate the cells *in vitro* (Ning et al., 2010). However, the *in vitro* proliferation capacity of SMCs is limited, hindering their application in the field of tissue engineering (Li et al., 2016).

Mesenchymal stem cells (MSCs) are a type of adult stem cells, which were first discovered in the bone marrow, and subsequently found in many kinds of tissues during the occurrence and development of the human body. They can differentiate not only into mesoderm, but can also differentiate into endoderm and neuroectoderm-derived cells, and can be targeted to differentiate into various terminal functional cells, such as muscle cells, osteocyte, vascular cells, endothelial cells, and nerve cells, under specific conditions (Galipeau and Sensebe, 2018). MSCs have obtained increasing attention as seed cells for tissue engineering (Gao et al., 2021; Rajasingh et al., 2021). In addition, MSCs can also promote the repair of various tissue injuries through direct intercellular contact and paracrine factors such as cytokines, growth factors, and small extracellular vesicles (sEV) (Keshtkar et al., 2018; Xia et al., 2019).

Umbilical cord mesenchymal stem cells (UCMSCs) are easy to collect, have strong proliferative ability and low immunogenicity, and that secreted sEV are often used in regenerative medicine and the treatment of various diseases (Ding et al., 2015; Can et al., 2017). UCMSC-derived sEV (UCMSC-sEV) are tiny vesicles with a diameter of 30–150 nm secreted by UCMSCs. As a carrier of intercellular communication cargo, UCMSC-sEV enter target cells and regulate a variety of physiological processes including cell proliferation, differentiation, migration and apoptosis (Fu et al., 2019; Keshtkar et al., 2018; Wu et al., 2018; Yaghoubi et al., 2019). However, whether UCMSC-sEV can promote the proliferation and migration of pediatric urethral smooth muscle cells (PUMSCs) and the related molecular mechanisms are still unclear. This study explored the potential molecular mechanisms that affect the functions of PUMSCs.

## Methods

### Isolation and Culture of PUSMCs

This experiment was approved by the Ethics Committee of the Maternal and Child Health Hospital of Foshan, and informed consent was obtained from the donors. Pediatric urethral tissues were collected from patients with hypospadias. The urethral tissues were cut into small pieces using surgical scissors and transferred to centrifuge tubes. A five-fold volume of 1 mg/ml type II collagenase (Sigma) was added into the tubes, and the tissue was digested at 37°C for 4 h. After filtration through a 70-mesh cell sieve (NEST, China), the cells were cultured in SMC complete medium (ScienCell, United States). When the cells were cultured to 80% confluency, they were digested with 0.25% trypsin (Gibco, United States) and seeded into a new culture dish. After the cells adhered to the dishes for 0.5–1 h, the supernatant was then transferred to a new culture dish, and the above process was repeated. Multiple passages of purified PUSMCs were obtained through this procedure.

### Human UCMSC Culture

UCMSCs were purchased from Cyagen Biosciences Inc. (Guangzhou, China) and were cultured in the complete MSC medium (Cyagen, China) supplemented with 10% fetal bovine serum (FBS). Cells were passaged at a ratio of 1:2.

### Cell Identification

The third and sixth passages of PUSMCs were seeded into 96-well plates. Six wells were stained with cell counting kit-8 (CCK-8) at the same time every day and then analyzed on a microplate reader (MK3, Thermo). The results were recorded, and a growth curve was plotted. After the sixth passage, the PUSMCs were washed, permeabilized and blocked with goat serum, and fluorescein isothiocyanate (FITC)-labeled α-smooth muscle actin (α-SMA) monoclonal antibody (Bioss, China) was added; the cells were incubated overnight in a 4°C refrigerator in the dark. Then, the cells were counterstained with 4′,6-diamidino-2-phenylindole (DAPI). The expression of SMA-α, a marker for SMCs, was observed under an inverted fluorescence microscope.

Surface markers of UCMSCs were detected by a flow cytometry assay. UCMSCs were incubated with fluorescently labeled CD29, CD44, CD45, CD73, CD90, and human leucocyte antigen DR (HLA-DR) monoclonal antibodies (Biolegend, United States) for half an hour, and the results were analyzed using a CytoFLEX flow cytometer (Beckman Coulter, United States).

### Extraction, Purification and Identification of sEV

The FBS used for cell culture was spun at 100,000 g overnight to remove the existing serum EVs. UCMSCs at passages four to eight were cultured in sEV-free medium for 48 h, and the cell supernatant was collected. sEV were isolated using ultracentrifugation and purified using a sEV purification reagent and concentration system (Exojuice, WeinaBio, China). Cell conditioned medium was collected and centrifuged at 300 g for 20 min to remove cells and centrifuged at 10,000 g for 20 min to remove cell debris. Supernatants were transferred into ultracentrifugation tubes and centrifuged at 100, 000 g for 70 min in order to obtain a pellet enriched in sEV. The precipitates were suspended in PBS and purified by Exojuice Kit at 100,000 g for 70 min in accordance with the protocol provided by the manufacturer. The resultant sEV was stored at −80°C.

sEV characterization was performed following the International Society for Extracellular Vesicles guidelines (Thery et al., 2018; Witwer et al., 2019). Briefly, the morphology of isolated sEV samples were visualized using transmission electron microscopy (TEM), and the particle size and concentration of sEV were analyzed by NanoFCM analysis. The sEV specific proteins, including CD9, CD63, CD81 and tumor susceptibility 101 (TSG101), were analyzed by Western blot.

### CCK-8 Assay

PUSMCs were seeded in 96-well plates at a density of 5,000 cells/well/100 µL. After 12 h of culture, medium containing UCMSC-sEV with or without PSB12379 (a CD73 inhibitor) was added to cells. After 24 and 48 h of culture, 10 μL of CCK-8 reagent was added, and the cells were incubated at 37°C for 1.5 h. Then a microplate reader (Thermo MK3, United States) was used to measure the absorbance at 450 nm.

### EdU Incorporation Assay

PUSMCs were seeded in 6-well plates at a density of 5×10^5^ cells/well. After 12 h of culture, medium containing UCMSC-sEV was added, and the culture was continued for 12 h. Then, the cells were treated with 5-ethynyl-2′-deoxyuridine (EdU) solution (Beyotime, China) for 2 h and then incubated in click reaction solution at 37°C for 30 min in the dark. The cells were stained following the instructions provided with the EdU solution and then imaged under a fluorescence microscope to calculate the percentage of EdU-positive cells.

### Scratch Wound Assay

A culture insert (Ibidi, Martinsried, Germany) was placed in the middle of a 24-well culture plate. Subsequently, PUSMCs were seeded at a density of 5×10^5^/ml per well (70 μL volume). After 24 h, the culture insert was carefully removed, and UCMSC-sEV were added to the PUSMCs for 12 h. Cell migration was imaged using an inverted microscope (Olympus IX 71, 100 × magnification, Olympus, Japan) and analyzed using ImageJ software v1.8 (National Institutes of Health, United States).

### Transwell Assay

PUSMCs were resuspended in serum-free medium and then were seeded in the upper chamber of a Transwell at a density of 5×10^5^ cells/ml (100 μL volume), and culture medium containing UCMSC-sEV with or without PSB12379 was added to the lower chamber of the Transwell. After 24 h of culture, the cells that had migrated to the lower chamber were counted after fixation and crystal violet staining.

### qRT–PCR Assay

Total RNA was extracted using TRIzol reagent (Thermo, United States), and cDNA was synthesized using HiScript^®^ III RT SuperMix for qPCR (+gDNA wiper) (Novvia, China). Quantitative PCR was performed using ChamQ universal SYBR qPCR Master Mix (Novenza, China) to detect the mRNA level of CD73. All gene sequences were obtained through GenBank, and primers were designed using Primer Premier five and synthesized by Sangong Biotech (Shanghai, China). The primer sequences are provided in Table 1.

**TABLE 1:** Primers used for real-time quantitative reverse transcription-polymerase chain reaction.

Gene	Strand	5–3′ Sequence
β-actin	Sense	GGC​ATC​CAC​GAA​ACT​ACA​TTC​AAT​TCC
	Anti-sense	GTA​CCA​CCA​GAC​AGC​ACT​GTG​TTG
CD 73	Sense	TGG​GAG​CTT​ACG​ATT​TTG​CAC​ACC
	Anti-sense	CGG​ATC​TGC​TGA​ACC​TTG​GTG​AAG

### Western Blotting

The protein concentration was determined using a bicinchoninic acid (BCA) reagent kit. Total protein (30 mg) was separated by 10% sodium dodecyl-sulfate polyacrylamide gel electrophoresis (SDS-PAGE) and transferred to a polyvinylidene difluoride (PVDF) membrane (Millipore, United States). The membrane was blocked with 5% skim milk for 2 h and then incubated with a primary antibody (dilution ratio of 1:1,000) at 4°C overnight. After washing with 0.1% Tween^®^ 20 (TBST) 3 times, the corresponding horseradish peroxidase-labeled secondary antibody (dilution ratio of 1:5,000) was added, and the membrane was incubated for 1 h. After washing with TBST 3 times, a chemiluminescence reagent (Tanon, China) was added dropwise to the membrane. Finally, the protein expression was observed using a chemiluminescent imaging system (Tanon, China). The monoclonal antibodies for CD9 and CD81 were from Affinity Biosciences (Affinity, United States). The monoclonal antibodies for TSG101 and CD63 were from Santa Cruz Biotechnology (Santa Cruz, United States). The monoclonal antibodies for CD73 were from Abcam (United Kingdom). The monoclonal antibodies for phospho-AKT, phospho-PI3K and glyceraldehyde-3-phosphate dehydrogenase (GAPDH) were from Bioss Biotechnology (Bioss, China).

### Immunofluorescence Staining

The extracted UCMSC-sEV were dissolved in phosphate buffered saline (PBS) containing 2% bovine serum albumin (BSA), followed by incubation with CD73 antibody to obtain phycoerythrin (PE)-conjugated CD73 sEV. Subsequently, the PE-conjugated CD73 sEV were incubated with PUSMCs at 37°C for 3 h, after which the cells were fixed in 4% paraformaldehyde for 15 min. The cytoskeleton was stained with FITC-phalloidin for 45 min, and the nuclei were stained with DAPI. Fluorescence microscopy was used to detect fluorescence signals in the cells.

### Adenosine Assay

PUSMCs were seeded at a density of 50,000 cells/well and cultured in medium with or without 5′AMP (Sigma) and UCMSC-sEV for 4 h. Then, the supernatant was collected and quickly frozen at −80°C. Adenosine concentrations were analyzed using an Adenosine Assay Kit (BioVision) in accordance with the protocol provided by the manufacturer. The fluorescent intensity was measured at Ex/Em 535/587 by Thermo Scientific Varioskan Flash Multimode Reader (Thermo, United States).

### Statistical Analysis

Shapiro-Wilk normality tests were performed for each set of data, in the case of normal distribution, the data are presented as mean ± SD. The data were assessed using the SPSS 11.0 program for Windows (SPSS Co., United States). Significance was evaluated at *p*-value of 0.05 and 0.01 using *t* test.

## Results

### Cell Identification

**FIGURE 1:**
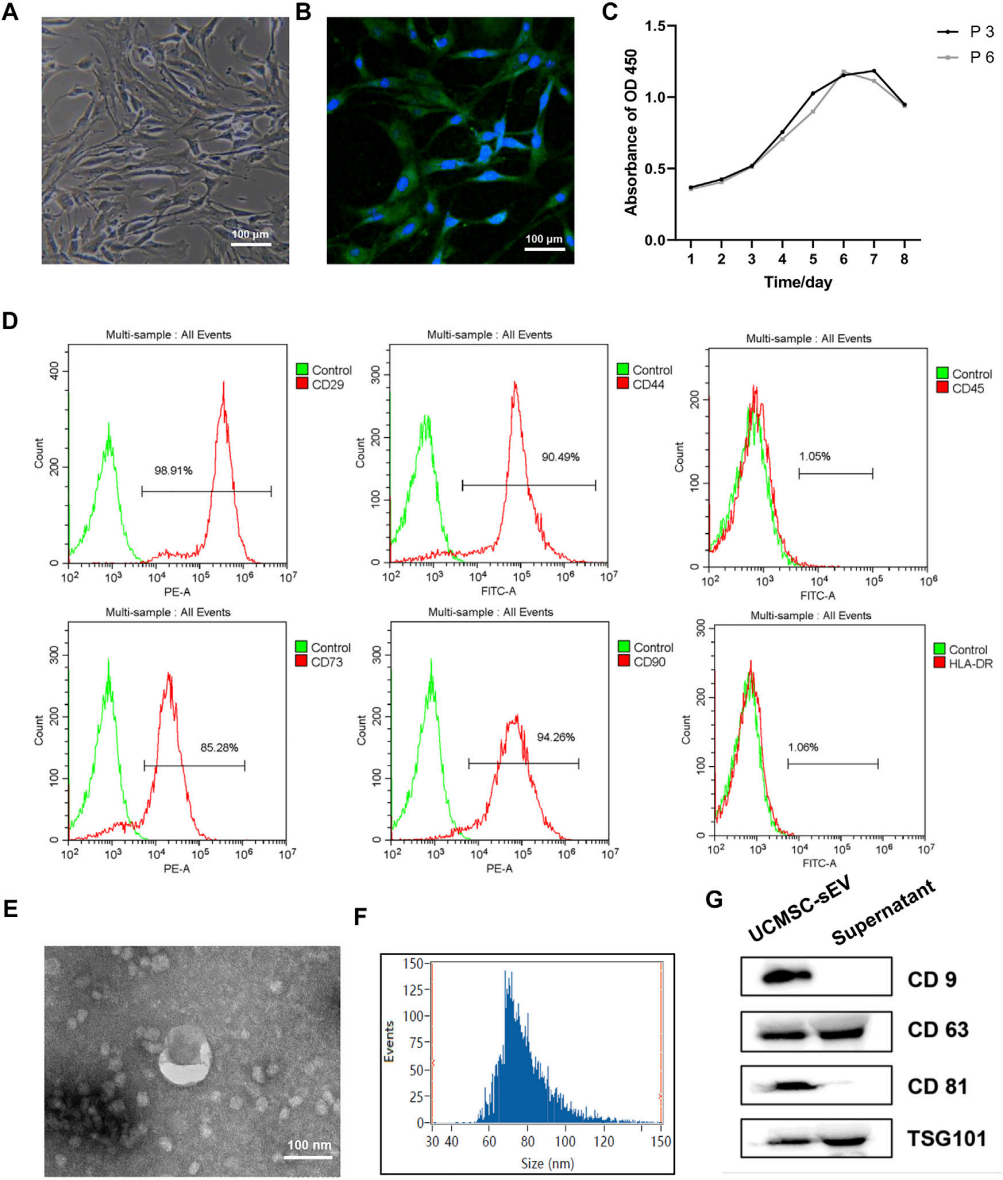
Identification of PUMSCs, UCMSCs and UCMSC-sEV. **(A)** Morphology of PUMSCs. **(B)** Immunofluorescence staining results show that the isolated PUMSCs express α-SMA, a smooth muscle cell surface marker. **(C)** Growth curve of PUMSCs. **(D)** Flow cytometric analysis of MSC surface markers shows that UCMSCs express high levels = of CD29, CD44, CD73, and CD90 but do not express CD45 and HLA-DR. **(E)** Transmission electron microscopy shows that the UCMSC-sEV are cup-shaped vesicles. Scale bar = 100 nm. **(F)** NanoFCM analysis of the particle size of UCMSC-sEV. **(G)** Western blotting was used to detect the expression of the sEV marker proteins CD9, CD63, CD81, and TSG101.

After passaging, the PUSMCs grew in bundles and exhibited a cascade arrangement and a “peak-to-valley” phenomenon (Figure 1A). The immunofluorescence assay results showed that the cultured cells expressed α-SMA, a smooth muscle surface marker (Figure 1B). The proliferation of PUSMCs was vigorous, and the growth curve (Figure 1C) trend was S-shaped, with lag, logarithmic and plateau phases.

Flow cytometry analysis of the surface markers of MSCs indicated that UCMSCs expressed high levels of the CD29, CD44, CD73, and CD90 but were negative for the CD45 and HLA-DR (Figure 1D), findings that are consistent with the general characteristics of MSCs.

### Identification of the sEV

Transmission electron microscopy revealed that the UCMSC-sEV were cup-shaped vesicles (Figure 1E). NanoFCM analysis indicated that the average diameter of the sEV was 78.91 nm and that the concentration was 2.53E+10 particles/mL (Figure 1F). Western blot results showed that the extracted sEV expressed CD81, CD63, CD9 and TSG101 (Figure 1G).

### UCMSC-sEV Promoted PUSMC Proliferation and Migration

**FIGURE 2:**
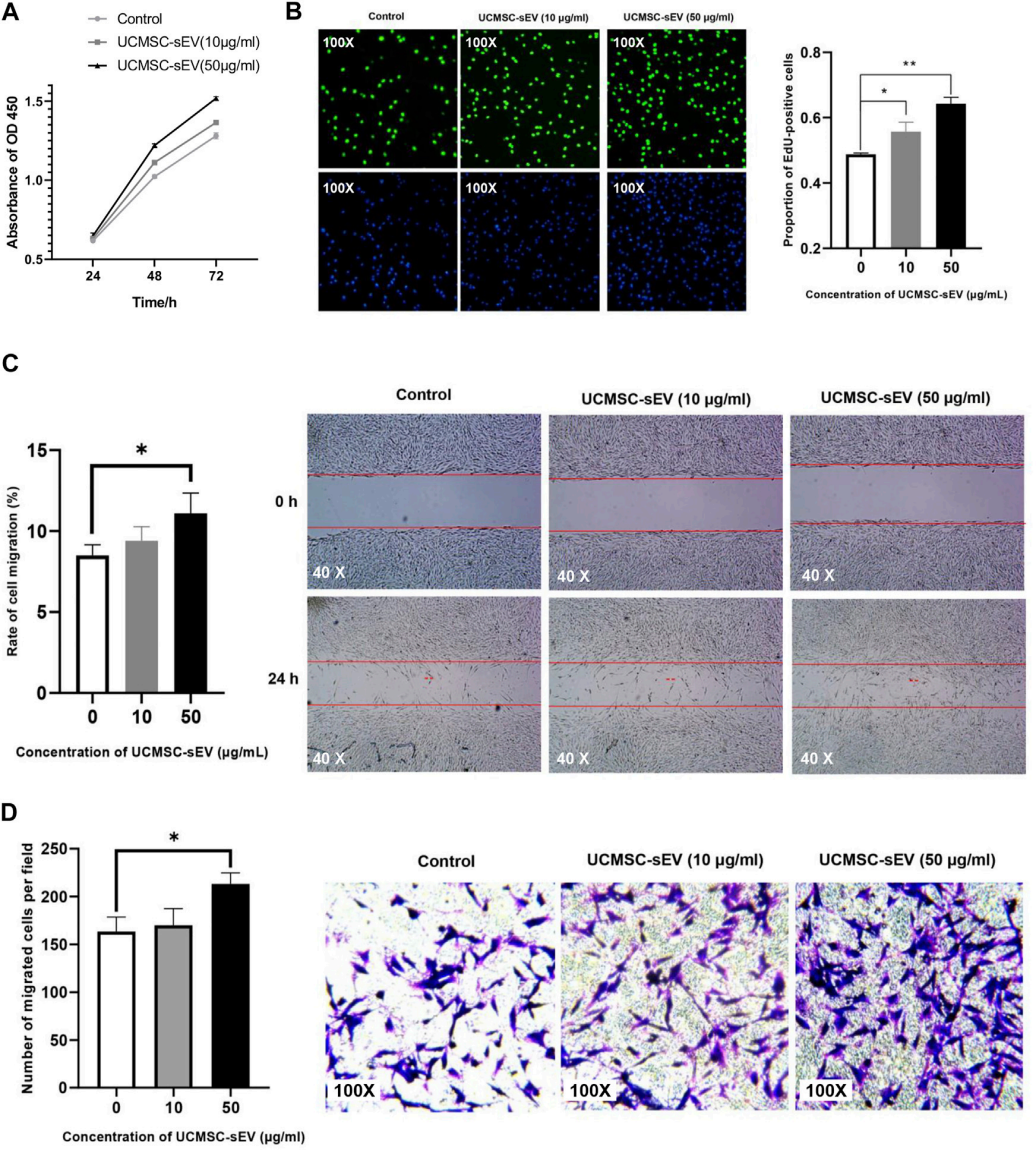
UCMSC-sEV promote the proliferation and migration of PUMSCs. **(A)** A CCK-8 assay was used to detect the proliferation of PUMSCs treated with 10 μg/ml and 50 μg/ml UCMSC-sEV for 24, 48 and 72 h **(B)** The EdU incorporation assay results show that UCMSC-sEV (10 μg/ml and 50 μg/ml) significantly promote the proliferation of PUMSCs. The scratch wound assay results **(C)** and Transwell assay results **(D)** are consistent. Low concentrations of UCMSC-sEV (10 μmol/L) have no effect on the migration of PUSMCs, and high concentrations of UCMSC-sEV (50 μmol/L) significantly promote the migration of PUSMCs.

Results of CCK-8 assays (Figure 2A) and EdU assays (Figure 2B) indicated that UCMSC-sEV promoted the proliferation of PUSMCs in a dose- and time-dependent manner.

As shown in Figures 2C,D, both scratch wound assay and Transwell assay results indicated that low concentrations of UCMSC-sEV (10 μmol/L) had no effect on the migration of PUSMCs, and high concentrations of UCMSC-sEV (50 μmol/L) significantly promoted the migration of PUSMCs.

### UCMSC-sEV Highly Expressed CD73 Molecules That Were Endocytosed by PUSMCs

**FIGURE 3:**
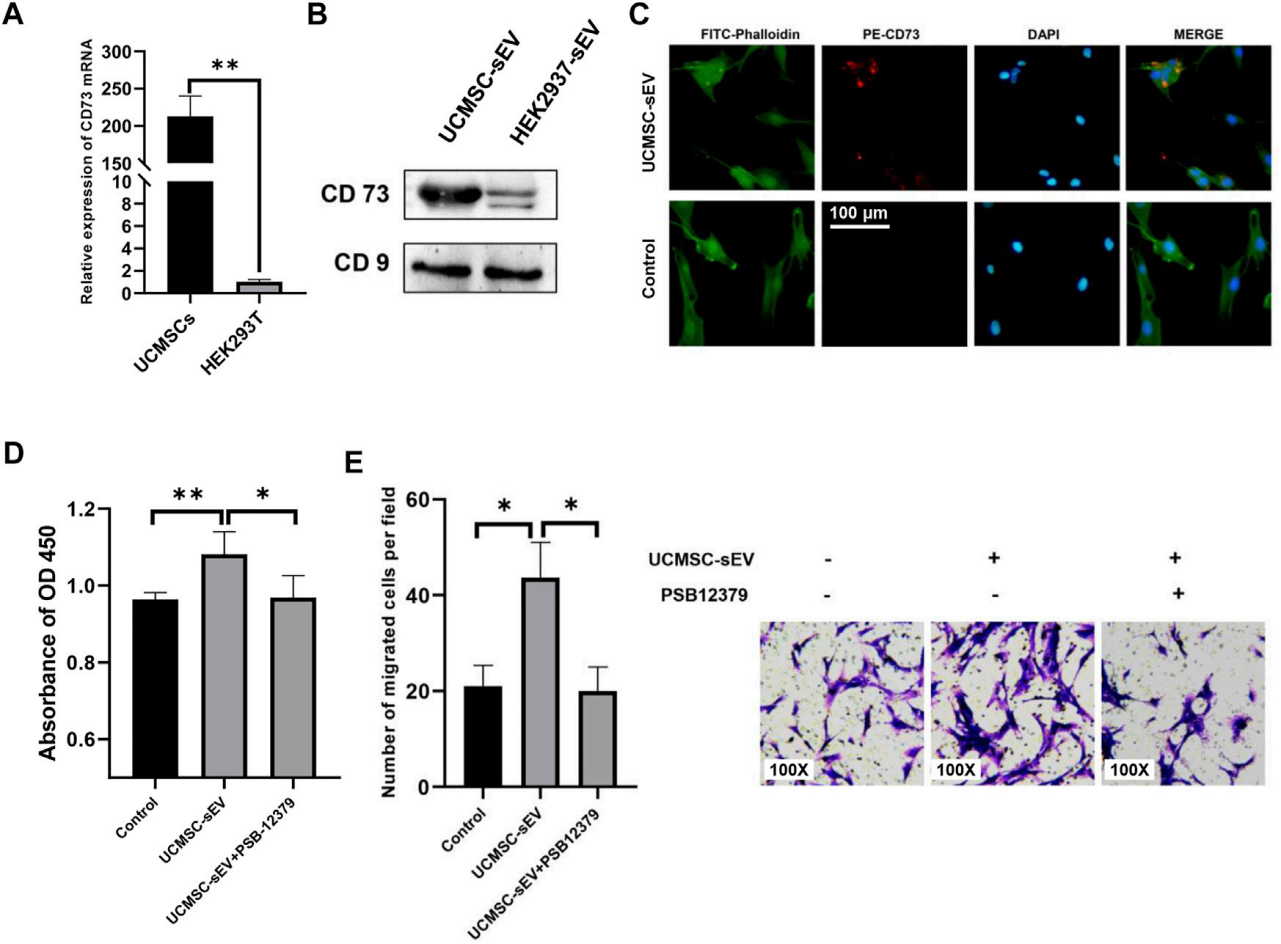
UCMSC-sEV promote proliferation and migration through CD73 molecules. **(A)** Detection of the mRNA expression level of CD73 in UCMSCs using real-time PCR (HEK293T was used as the control group). **(B)** Western blotting was used to detect the protein expression level of CD73 in UCMSC-sEV (HEK293T-sEV were used as the control group). **(C)** Immunofluorescence staining shows that PE-CD73-labeled sEV are internalized by PUMSCs. **(D)** CCK-8 and Transwell assay results **(E)** show that the inhibition of the sEV CD73 molecular activity by PSB12379 blocks the proliferation- and migration-promoting effects of UCMSC-sEV.

CD73, also known as ecto-5′-nucleotidase, is a cell surface enzyme that is highly expressed on the surface of UCMSCs (Figure 1D). To verify whether UCMSC-sEV also expressed CD73, qRT-PCR and Western blotting were used to assess the mRNA and protein levels of CD73 in UCMSCs and UCMSC-sEV, respectively. The results showed that compared with HEK293T cell-derived sEV (HEK293T-sEV), UCMSCs and UCMSC-sEV highly expressed CD73 mRNA and protein (Figure 3A and Figure 3B).

To confirm internalization of CD73-positive UCMSC-sEV by PUSMCs, a PE-conjugated anti-CD73 monoclonal antibody was used to label UCMSC-sEV. The labeled sEV were cocultured with PUSMCs for 3 h, and the internalization of UCMSC-sEV was observed under a fluorescence microscope. The red fluorescence of CD73 on the surface of UCMSC-sEV was localized to PUSMCs, indicating that CD73-positive UCMSC-sEV were endocytosed by PUSMCs (Figure 3C).

### Inhibition of CD73 Molecular Activity Blocked the Proliferation- and Migration-Promoting Effects of UCMSC-sEV

To further investigate whether the proliferation- and migration-promoting effects of UCMSC-sEV were related to the transmission of CD73 molecules, we added a CD73 inhibitor (PSB12379, MCE) to the medium contained with UCMSC-sEV. The results indicated that the addition of PSB12379 inhibited CD73 activity and blocked the proliferation- and migration-promoting effects of UCMSC-sEV on PUSMCs (Figures 3D,E).

### The CD73 Metabolite Adenosine Promotes the Proliferation and Migration of PUSMCs

**FIGURE 4:**
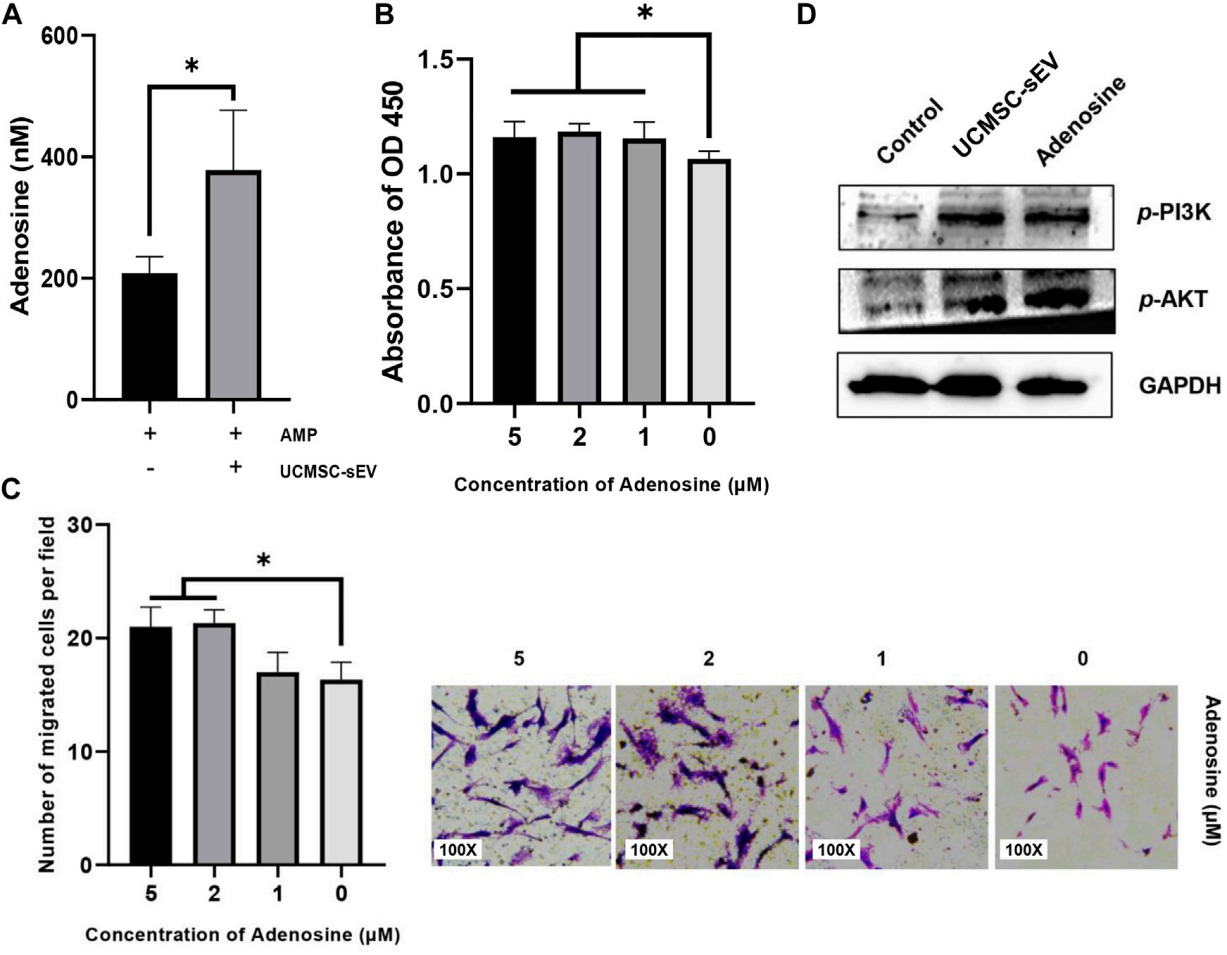
The sEV CD73 catalyzes the production of adenosine and activates the PI3K/AKT pathway to promote proliferation and migration. **(A)** UCMSC-sEV use exogenous 5′AMP to produce adenosine through surface CD73. **(B)** CCK-8 and Transwell assay results **(C)** show that the addition of exogenous adenosine significantly promotes the proliferation and migration of PUSMCs *in vitro*. **(D)** Western blot detection of the expression levels of PI3K/AKT in PUSMCs.

The expression of adenosine was detected in the cell culture supernatant containing 5′AMP (Sigma) and UCMSC-sEV (Figure 4A), indicating that CD73 on the surface of UCMSC-sEV used exogenous 5′AMP to produce adenosine. Moreover, the addition of exogenous adenosine to the culture medium of PUSMCs significantly promoted the proliferation and migration of PUSMCs (Figures 4B,C). These results demonstrate that the proliferation- and migration-promoting effects of UCMSC-sEV were related to the CD73/adenosine signaling axis.

### Activation of the PI3K/AKT Signaling Pathway

The PI3K/AKT signaling pathway is an important pathway for cell proliferation and survival and is closely related to adenosine metabolism. Therefore, we investigated the downstream PI3K/AKT signaling pathway by which CD73 affects the cellular functions of PUSMCs through Western blotting analysis. The protein expression levels of phospho-AKT and phospho-PI3K in PUSMCs increased after treated with adenosine or UCMSC-sEV (Figure 4D), indicating that UCMSC-sEV activated the PI3K/AKT signaling pathway in PUSMCs.

## Discussion

This study explored whether UCMSC-sEV could affect the proliferation and migration of PUSMCs and preliminarily researched the related molecular mechanism. The results showed that UCMSC-sEV significantly promoted the proliferation and migration of PUSMCs *in vitro* and this process may be related to the CD73/adenosine signaling axis and the PI3K/AKT pathway.

sEV are secreted and released by cells. As a carrier of intercellular communication, sEV has recently been confirmed as a new mechanism of cell-to-cell communication (Meldolesi, 2018; Kalluri and LeBleu, 2020; Rezaie et al., 2021). sEV are nanoscale lipid inclusion structures that contain substances such as proteins, mRNAs, and microRNAs and can accelerate the repair of damaged tissues by transferring RNA to adjacent target cells (Zhang et al., 2015; Kilchert et al., 2016; Hassanpour et al., 2020). Activated proteins can also be transferred to target cells by sEV and produce corresponding biological effects (Li et al., 2017; Toh et al., 2018). In this study, we first isolated UCMSC-sEV by the ultracentrifugation method and then characterized UCMSC-sEV by transmission electron microscopy, nanoflow cytometry, and the detection of the expression of the sEV marker molecules such as CD9, CD63, CD81, and TSG101, respectively.

In recent years, mesenchymal stem cell-derived sEV have been found to promote the proliferation and migration of many different types of cells *in vitro*, such as chondrocytes (Wen et al., 2022), vascular endothelial cells (Zhang et al., 2021), neuronal cells (Wei et al., 2020), keratinocytes (Kim et al., 2018), and fibroblasts (Shabbir et al., 2015). In view of the key role of sEV in boosting the regeneration and repair of different tissues, we speculate that UCMSC-sEV may also affect the repair of urethral tissue injury by affecting the biological behaviors of PUSMCs (such as proliferation and migration). In this study, in the presence of UCMSC-sEV, the *in vitro* migration and proliferation rates of PUSMCs were significantly higher than those of PUSMCs in the negative control group. These results demonstrate that UCMSC-sEV can indeed increase the proliferation and migration of PUSMCs *in vitro*, the finding that is consistent with the report by Wei (Huo et al., 2020) that bone marrow mesenchymal stem cell-derived sEV carrying miR-21-5p promote the proliferation of corpus cavernosum SMCs and inhibit their apoptosis *in vitro*.

As a surface marker of MSCs, CD73 can catalyze the hydrolysis of adenosine 5-phosphate to adenosine and exert a series of biological functions through the interaction of adenosine and adenosine receptors (Adamiak et al., 2019). Studies have shown that CD73 ^+^ MSCs are the dominant subpopulation involved in myocardial repair (Li et al., 2021). CD73 is over-expressed in tumor tissues and can increase tumor cell proliferation (Xie et al., 2017). During tumorigenesis and development, CD73 promotes the formation of new blood vessels by endothelial cells (Wang et al., 2013). This study found that UCMSCs highly expressed CD73 and could carry CD73 molecules to PUSMCs through the communication function of sEV to generate adenosine, thereby affecting cell proliferation and migration. When using PSB12379 to inhibit the CD73 enzymatic activity of UCMSC-sEV, the proliferation- and migration-promoting activities of UCMSC-sEV were no longer observed. To determine the signaling pathways through which the proliferation- and migration-promoting effects of UCMSC-sEV are mediated, we hypothesized that PI3K/AKT is a possible candidate pathway because the PI3K/AKT pathway is closely related to cell proliferation and migration and exosomal CD73 catalyzes adenosine production, and after binding to adenosine receptors, downstream signaling pathways, such as AKT and ERK, can be activated (Chew et al., 2019; Ma et al., 2019). In this study, we demonstrated that UCMSC-sEV and adenosine-mediated cell proliferation and migration of PUSMCs could indeed cause the phosphorylation of PI3K and AKT.

In summary, this study demonstrated that UCMSC-sEV can significantly promote PUSMC proliferation and migration *in vitro*. Moreover, we also found that UCMSC-sEV increased the proliferation and migration of PUSMCs *in vitro* through the activation of the CD73/adenosine signaling axis and the PI3K/AKT pathway.

## Data Availability

The original contributions presented in the study are included in the article/supplementary materials, further inquiries can be directed to the corresponding author.

## References

[B1] Abbas T. O. Mahdi E. Hasan A. AlAnsari A. Pennisi C. P. (2017). Current Status of Tissue Engineering in the Management of Severe Hypospadias. Front. Pediatr. 5, 283. 10.3389/fped.2017.00283 29404308 PMC5786532

[B2] Adamiak M. Bujko K. Brzezniakiewicz-Janus K. Kucia M. Ratajczak J. Ratajczak M. Z. (2019). The Inhibition of CD39 and CD73 Cell Surface Ectonucleotidases by Small Molecular Inhibitors Enhances the Mobilization of Bone Marrow Residing Stem Cells by Decreasing the Extracellular Level of Adenosine. Stem Cel Rev Rep 15 (6), 892–899. 10.1007/s12015-019-09918-y PMC6925070 31520298

[B3] Arenas da Silva L. F. Micol L. Tiemessen D. van Kuppevelt T. H. Frey P. Oosterwijk E. (2014). Is There a Need for Smooth Muscle Cell Transplantation in Urethral Reconstruction? Tissue Eng. A 20 (9-10), 1542–1549. 10.1089/ten.TEA.2013.0185 24329538

[B4] Can A. Celikkan F. T. Cinar O. (2017). Umbilical Cord Mesenchymal Stromal Cell Transplantations: A Systemic Analysis of Clinical Trials. Cytotherapy 19 (12), 1351–1382. 10.1016/j.jcyt.2017.08.004 28964742

[B5] Chan Y. Y. Bury M. I. Yura E. M. Hofer M. D. Cheng E. Y. Sharma A. K. (2020). The Current State of Tissue Engineering in the Management of Hypospadias. Nat. Rev. Urol. 17 (3), 162–175. 10.1038/s41585-020-0281-4 32024995

[B6] Chew J. R. J. Chuah S. J. Teo K. Y. W. Zhang S. Lai R. C. Fu J. H. (2019). Mesenchymal Stem Cell Exosomes Enhance Periodontal Ligament Cell Functions and Promote Periodontal Regeneration. Acta Biomater. 89, 252–264. 10.1016/j.actbio.2019.03.021 30878447

[B7] Ding D.-C. Chang Y.-H. Shyu W.-C. Lin S.-Z. (2015). Human Umbilical Cord Mesenchymal Stem Cells: A new era for Stem Cell Therapy. Cel Transpl. 24 (3), 339–347. 10.3727/096368915X686841 25622293

[B8] Fu X. Liu G. Halim A. Ju Y. Luo Q. Song A. G. (2019). Mesenchymal Stem Cell Migration and Tissue Repair. Cells 8 (8), 784. 10.3390/cells8080784 PMC6721499 31357692

[B9] Galipeau J. Sensébé L. (2018). Mesenchymal Stromal Cells: Clinical Challenges and Therapeutic Opportunities. Cell Stem Cell 22 (6), 824–833. 10.1016/j.stem.2018.05.004 29859173 PMC6434696

[B10] Gao G. Fan C. Li W. Liang R. Wei C. Chen X. (2021). Mesenchymal Stem Cells: Ideal Seeds for Treating Diseases. Hum. Cel 34 (6), 1585–1600. 10.1007/s13577-021-00578-0 PMC8284686 34272720

[B11] Hassanpour M. Rezabakhsh A. Rezaie J. Nouri M. Rahbarghazi R. (2020). Exosomal Cargos Modulate Autophagy in Recipient Cells via Different Signaling Pathways. Cell Biosci 10, 92. 10.1186/s13578-020-00455-7 32765827 PMC7395405

[B12] Huo W. Li Y. Zhang Y. Li H. (2020). Mesenchymal Stem Cells‐derived Exosomal microRNA‐21‐5p Downregulates PDCD4 and Ameliorates Erectile Dysfunction in a Rat Model of Diabetes Mellitus. FASEB j. 34 (10), 13345–13360. 10.1096/fj.202000102RR 32808325

[B13] Kalluri R. LeBleu V. S. (2020). The Biology , Function , and Biomedical Applications of Exosomes. Science 367 (6478), eaau6977. 10.1126/science.aau6977 32029601 PMC7717626

[B14] Keshtkar S. Azarpira N. Ghahremani M. H. (2018). Mesenchymal Stem Cell-Derived Extracellular Vesicles: Novel Frontiers in Regenerative Medicine. Stem Cel Res. Ther. 9 (1), 63. 10.1186/s13287-018-0791-7 PMC5845209 29523213

[B15] Kilchert C. Wittmann S. Vasiljeva L. (2016). The Regulation and Functions of the Nuclear RNA Exosome Complex. Nat. Rev. Mol. Cel Biol 17 (4), 227–239. 10.1038/nrm.2015.15 26726035

[B16] Kim S. Lee S. Kim H. Kim T. (2018). Exosomes Secreted from Induced Pluripotent Stem Cell-Derived Mesenchymal Stem Cells Accelerate Skin Cell Proliferation. Ijms 19 (10), 3119. 10.3390/ijms19103119 PMC6213597 30314356

[B17] Li Q. Hou H. Li M. Yu X. Zuo H. Gao J. (2021). CD73+ Mesenchymal Stem Cells Ameliorate Myocardial Infarction by Promoting Angiogenesis. Front. Cel Dev. Biol. 9, 637239. 10.3389/fcell.2021.637239 PMC8152667 34055772

[B18] Li W. Li C. Zhou T. Liu X. Liu X. Li X. (2017). Role of Exosomal Proteins in Cancer Diagnosis. Mol. Cancer 16 (1), 145. 10.1186/s12943-017-0706-8 28851367 PMC5576100

[B19] Li Y. Wen Y. Wang Z. Wei Y. Wani P. Green M. (2016). Smooth Muscle Progenitor Cells Derived from Human Pluripotent Stem Cells Induce Histologic Changes in Injured Urethral Sphincter. Stem Cell Transl Med 5 (12), 1719–1729. 10.5966/sctm.2016-0035 PMC5189655 27460854

[B20] Ma X.-L. Shen M.-N. Hu B. Wang B.-L. Yang W.-J. Lv L.-H. (2019). CD73 Promotes Hepatocellular Carcinoma Progression and Metastasis via Activating PI3K/AKT Signaling by Inducing Rap1-Mediated Membrane Localization of P110β and Predicts Poor Prognosis. J. Hematol. Oncol. 12 (1), 37. 10.1186/s13045-019-0724-7 30971294 PMC6458749

[B21] Meldolesi J. (2018). Exosomes and Ectosomes in Intercellular Communication. Curr. Biol. 28 (8), R435–R444. 10.1016/j.cub.2018.01.059 29689228

[B22] Ning N. Lin G. Lue T. F. Lin C.-S. (2010). Effects of Estrogen, Raloxifene, and Levormeloxifene on the Expression of Rho-Kinase Signaling Molecules in Urethral Smooth Muscle Cells. Urology 76 (6), e6–1517. 10.1016/j.urology.2010.07.470 PMC3537261 20970835

[B23] Rajasingh S. Sigamani V. Selvam V. Gurusamy N. Kirankumar S. Vasanthan J. (2021). Comparative Analysis of Human Induced Pluripotent Stem Cell-Derived Mesenchymal Stem Cells and Umbilical Cord Mesenchymal Stem Cells. J. Cel. Mol. Med. 25 (18), 8904–8919. 10.1111/jcmm.16851 PMC8435459 34390186

[B24] Rezaie J. Aslan C. Ahmadi M. Zolbanin N. M. Kashanchi F. Jafari R. (2021). The Versatile Role of Exosomes in Human Retroviral Infections: From Immunopathogenesis to Clinical Application. [Journal Article; Review]. Cel Biosci 11 (1), 19. 10.1186/s13578-021-00537-0 PMC7810184 33451365

[B25] Sergeant G. P. Hollywood M. A. Thornbury K. D. (2019). Spontaneous Activity in Urethral Smooth Muscle. Adv. Exp. Med. Biol. 1124, 149–167. 10.1007/978-981-13-5895-1_6 31183826

[B26] Shabbir A. Cox A. Rodriguez-Menocal L. Salgado M. Van Badiavas E. (2015). Mesenchymal Stem Cell Exosomes Induce Proliferation and Migration of normal and Chronic Wound Fibroblasts, and Enhance Angiogenesis *In Vitro* . Stem Cell Dev 24 (14), 1635–1647. 10.1089/scd.2014.0316 PMC4499790 25867197

[B27] Thery C. Witwer K. W. Aikawa E. Alcaraz M. J. Anderson J. D. Andriantsitohaina R. (2018). Minimal Information for Studies of Extracellular Vesicles 2018 (MISEV2018): A Position Statement of the International Society for Extracellular Vesicles and Update of the MISEV2014 Guidelines. [Journal Article]. J. Extracell Vesicles 7 (1), 1535750. 10.1080/20013078.2018.1535750 30637094 PMC6322352

[B28] Toh W. S. Lai R. C. Zhang B. Lim S. K. (2018). MSC Exosome Works through a Protein-Based Mechanism of Action. Biochem. Soc. Trans. 46 (4), 843–853. 10.1042/BST20180079 29986939 PMC6103455

[B29] Wang L. Tang S. Wang Y. Xu S. Yu J. Zhi X. (2013). Ecto-5'-nucleotidase (CD73) Promotes Tumor Angiogenesis. Clin. Exp. Metastasis 30 (5), 671–680. 10.1007/s10585-013-9571-z 23508889

[B30] Wei H. Xu Y. Chen Q. Chen H. Zhu X. Li Y. (2020). Mesenchymal Stem Cell-Derived Exosomal miR-223 Regulates Neuronal Cell Apoptosis. Cell Death Dis 11 (4), 290. 10.1038/s41419-020-2490-4 32341353 PMC7184756

[B31] Wen C. Lin L. Zou R. Lin F. Liu Y. (2022). Mesenchymal Stem Cell-Derived Exosome Mediated Long Non-coding RNA KLF3-AS1 Represses Autophagy and Apoptosis of Chondrocytes in Osteoarthritis. Cell Cycle 21 (3), 289–303. 10.1080/15384101.2021.2019411 34964696 PMC8855872

[B32] Witwer K. W. Van Balkom B. Bruno S. Choo A. Dominici M. Gimona M. (2019). Defining Mesenchymal Stromal Cell (MSC)-derived Small Extracellular Vesicles for Therapeutic Applications. [Journal Article]. J. Extracell Vesicles 8 (1), 1609206. 10.1080/20013078.2019.1609206 31069028 PMC6493293

[B33] Wu P. Zhang B. Shi H. Qian H. Xu W. (2018). MSC-exosome: A Novel Cell-free Therapy for Cutaneous Regeneration. Cytotherapy 20 (3), 291–301. 10.1016/j.jcyt.2017.11.002 29434006

[B34] Xia J. Minamino S. Kuwabara K. Arai S. (2019). Stem Cell Secretome as a New Booster for Regenerative Medicine. Biosci. Trends. 13 (4), 299–307. 10.5582/bst.2019.01226 31527327

[B35] Xie M. Qin H. Luo Q. Huang Q. He X. Yang Z. (2017). MicroRNA-30a Regulates Cell Proliferation and Tumor Growth of Colorectal Cancer by Targeting CD73. BMC Cancer 17 (1), 305. 10.1186/s12885-017-3291-8 28464916 PMC5414330

[B36] Yaghoubi Y. Movassaghpour A. Zamani M. Talebi M. Mehdizadeh A. Yousefi M. (2019). Human Umbilical Cord Mesenchymal Stem Cells Derived-Exosomes in Diseases Treatment. Life Sci. 233, 116733. 10.1016/j.lfs.2019.116733 31394127

[B37] Zhang J. Li S. Li L. Li M. Guo C. Yao J. (2015). Exosome and Exosomal microRNA: Trafficking, Sorting, and Function. Genomics Proteomics Bioinformatics 13 (1), 17–24. 10.1016/j.gpb.2015.02.001 25724326 PMC4411500

[B38] Zhang Y. Xie Y. Hao Z. Zhou P. Wang P. Fang S. (2021). Umbilical Mesenchymal Stem Cell-Derived Exosome-Encapsulated Hydrogels Accelerate Bone Repair by Enhancing Angiogenesis. ACS Appl. Mater. Inter. 13 (16), 18472–18487. 10.1021/acsami.0c22671 33856781

